# PD15 - MP29-02 effectively relieves both nasal and ocular symptoms in adolescents aged 12-17 years: results from a meta-analysis of 4 randomised controlled Seasonal Allergic Rhinitis (SAR) trials

**DOI:** 10.1186/2045-7022-4-S1-P15

**Published:** 2014-02-28

**Authors:** Erkka Valovirta, Ruth Murray, Ullrich Munzel, Nikos Papadopoulos

**Affiliations:** 1 Suomen Terveystalo Allergy Clinic, Turku, Finland; 2 Medscript, Dundalk, Ireland; 3 Meda, Bad Homburg, Germany; 4 University of Athens, Athens, Greece

## Introduction

Two large paediatric studies are currently underway in the U.S. to investigate efficacy and safety of MP29-02 (Dymista) in children, with a view to extending its indication to those aged ≥6 yrs. In line with FDA recommendations, these studies compare MP29-02 to placebo. To mimic the clinical trial design of these on-going pediatric studies, this meta-analysis assessed all data available for moderate-to-severe SAR patients aged 12-17 years, who received either MP29-02 or placebo in the same vehicle and device.

## Methods

Data from 4 multi-centre, parallel-group, randomized, double-blind, placebo-controlled, 14-day studies were pooled. A total of 97 patients aged 12-17 yrs received MP29-02 (a novel intranasal formulation of azelastine hydrochloride and fluticasone propionate) 1 spray/nostril bid (total daily dose: AZE 548µg; FP 200µg) and 112 patients received placebo spray 1 spray/nostril bid. The primary efficacy variable was change from baseline over 14-days in reflective total nasal symptom score (rTNSS; AM +PM; MAX=24), the sum of 4 symptom scores for congestion, itching, rhinorrhoea and sneezing. Reflective total ocular symptom score (rTOSS; AM + PM; Max = 18) was an important secondary endpoint.

## Results

Patients aged 12-17 yrs treated with MP29-02 experienced a 4.29 point mean improvement from baseline (18.7 [SD 2.7]) in their rTNSS, significantly more the 2.06 point improvement from baseline (18.7 [SD 2.8]) observed in those treated with placebo (diff: 2.23; 95% CI: 3.23; 1.22; p<0.0001). Similarly, in this adolescent patient population treatment with MP29-02 produced a significant mean improvement in the rTOSS of 2.23 points from baseline (11.2 [SD 4.4]) compared to 1.04 points from baseline (10.9 [SD 4.0]) with placebo (diff: 1.19; 95% CI: 2.06, 0.32; p=0.0080).

## Conclusion

These results show that adolescent SAR patients treated with MP29-02 experience significant relief from both their nasal and ocular symptoms. Similar beneficial effects may be expected in pediatric patients.

